# Efficacy and Safety of Fruquintinib Plus TAS‐102 in Patients With Previously Treated Metastatic Colorectal Cancer: Results From a Single‐Arm, Multi‐Center, Phase II Study

**DOI:** 10.1002/mco2.71070

**Published:** 2026-10-13

**Authors:** Jianjun Peng, Yong Li, Ling Huang, Jianhua Liu, Jianjun Xiao, Derong Xie, Wei Wang, Zongcheng Zhang, Chengyin Weng, Xiaoshu Chai, Huifen Li, Xiaomei Jiang, Yan Qian, Risheng Zhao, Sile Chen, Zhimin Jiang, Lingdan Le, Xueqin Li, Jianting Long, Haibo Zhang, Chuangqi Chen, Shirong Cai

**Affiliations:** ^1^ The First Affiliated Hospital of Sun Yat‐sen University Guangzhou People's Republic of China; ^2^ Guangdong Provincial Hospital of Chinese Medicine Zhuhai People's Republic of China; ^3^ Guangdong Provincial People's Hospital, Guangdong Academy of Medical Sciences Southern Medical University Guangzhou People's Republic of China; ^4^ Zhongshan City People's Hospital Zhongshan People's Republic of China; ^5^ Sun Yat‐sen Memorial Hospital of Sun Yat‐sen University Guangzhou People's Republic of China; ^6^ The First People's Hospital of Foshan Foshan People's Republic of China; ^7^ Liwan Central Hospital of Guangzhou Guangzhou People's Republic of China; ^8^ Guangzhou First People's Hospital Guangzhou People's Republic of China; ^9^ Guangdong Provincial Hospital of Chinese Medicine Guangzhou People's Republic of China; ^10^ Shenshan Medical Center Sun Yat‐sen Memorial Hospital, Sun Yat‐sen University Shanwei People's Republic of China

**Keywords:** fruquintinib, mCRC, TAS‐102, third‐line

## Abstract

The clinical efficacy of currently available third‐ and later‐line treatment regimens for patients with metastatic colorectal cancer (mCRC) remains suboptimal. This study was designed to investigate fruquintinib plus TAS‐102 regimen in this patient population. This study was a prospective, multi‐center, single‐arm phase II trial. A total of 50 patients with mCRC who had experienced treatment failure to at least two standard therapies were enrolled. The primary endpoint was progression‐free survival (PFS) and secondary endpoints included overall survival (OS), objective response rate (ORR), disease control rate (DCR), and safety. Survival outcomes were estimated using the Kaplan–Meier method. With a median follow‐up duration of 25.1 months, the median PFS was 6.07 months (95% CI: 4.07–7.87) and the median OS was 14.10 months (95% CI: 9.84–23.28). The ORR was 10.0% (95% CI: 3.3–21.8), with a DCR of 66.0% (95% CI: 51.2–78.8). All the patients experienced treatment‐related adverse events (TRAEs), with 68.0% reporting grade ≥ 3 TRAEs. No serious adverse events (AEs), deaths, or new safety concerns were reported. In conclusion, fruquintinib combined with TAS‐102 showed preliminary anti‐tumor activity with a manageable safety profile in patients with refractory mCRC, which highlights its potential as a viable later‐line therapeutic option.

**Trial Registration**: NCT05004831

## Graphical abstract

This study is the first prospective clinical trial that evaluated the efficacy and safety of fruquintinib combined with TAS‐102 in the later‐line treatment of patients with mCRC. Fruquintinib plus TAS‐102 demonstrated promising efficacy and manageable safety profile in patients with mCRC who had failed at least two standard therapies, supporting its potential as a treatment option in this setting.

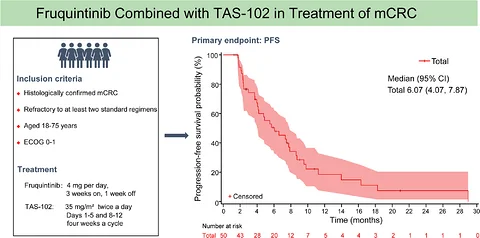

## 1 Introduction

According to data reported by the National Cancer Center in 2024, there were approximately 517,100 new colorectal cancer (CRC) cases in China in 2022, and CRC ranks second in incidence among all malignant tumors. Its incidence showed an increasing trend between 2000 and 2018, with a more notable increase observed in males [1]. Unlike developed countries in Europe and America, where early‐stage diagnoses are more prevalent, in China, approximately a quarter of patients with CRC are diagnosed with metastatic disease at initial presentation [2]. For these patients, systemic drug therapy serves as the primary treatment strategy. Currently, first‐line and second‐line treatments for metastatic CRC (mCRC) primarily consist of chemotherapy—combining fluoropyrimidines with oxaliplatin and/or irinotecan—along with targeted agents. These targeted therapies include vascular endothelial growth factor (VEGF) inhibitors or epidermal growth factor receptor (EGFR) inhibitors, with the latter targeting patients with left‐sided and *RAS/BRAF* wild‐type colon cancer. The continuous emergence of novel agents and the implementation of individualized treatment plans have significantly improved the efficacy and safety of first‐ and second‐line treatments for mCRC patients, allowing more patients to proceed with treatment after second‐line progression.

According to the *Blue Paper on the Current Status of Clinical Diagnosis and Treatment of mCRC*, which was released by the Colorectal Cancer Committee of the Chinese Society of Clinical Oncology, in 2023, approximately 58.2% of patients with CRC were eligible for third‐ or later‐line treatment after progression on first‐ and second‐line therapies [3]. Most of these patients maintain good performance status and can tolerate treatment of a certain intensity. On the basis of evidence‐based medicine, most national and regional treatment guidelines recommend monotherapies, such as regorafenib (CONCUR and CORRECT studies), fruquintinib (FRESCO and FRESCO2 studies), or trifluridine/tipiracil (TAS‐102; RECOURSE and TERRA studies) for third‐line and beyond settings. However, the clinical efficacies of these regimens remain limited; notably, treated patients have a progression‐free survival (PFS) time of approximately 2–4 months, a median overall survival (OS) of 5–8 months, and a low objective response rate (ORR) of only 1%–4% [4, 5, 6, 7, 8, 9].

Fruquintinib is a potent, highly selective small‐molecule inhibitor of VEGFR‐1, ‐2, and ‐3 that blocks the growth of new blood vessels, which are associated with tumor proliferation [10]. TAS‐102, an oral compound composed of trifluridine (FTD) and tipiracil (TPI) at a 1:0.5 molar ratio, exerts its anticancer effects through the following three distinct pathways: TPI inhibits thymidine phosphorylase (TP)‐mediated degradation of FTD, thereby maintaining FTD concentrations in the blood and increasing its antitumor activity; FTD suppresses thymidylate synthase (TS) activity, blocks the uracil‐to‐thymidine pathway, disrupts DNA synthesis in tumor cells, and induces apoptosis; additionally, FTD is incorporated into DNA in place of thymine during replication, disrupting DNA function [11]. Building on the evidence from the TERRA and FRESCO trials, both TAS‐102 and fruquintinib as monotherapy regimens have obtained regulatory approval in China for the treatment of mCRC patients, who have previously undergone fluoropyrimidine‐, oxaliplatin‐, and irinotecan‐based chemotherapy, as well as individuals who have received anti‐VEGF or anti‐EGFR therapies (for *RAS* wild‐type tumors) or are deemed ineligible for such treatments.

The phase III SUNLIGHT trial provided evidence that the combination of TAS‐102 and bevacizumab yielded a significant improvement in PFS and OS in patients with refractory mCRC, with a manageable safety profile and acceptable tolerability [12]. However, this study did not enroll Asian patients. In the phase III ROBiTS study, which explored TAS‐102 combined with bevacizumab in the Japanese population, the combination significantly prolonged PFS to 4.0 months, while the OS was even shorter than that in the TAS‐102 monotherapy [13]. Notably, long‐term bevacizumab treatment inevitably leads to resistance. In the phase III FRESCO trial, fruquintinib improved OS and PFS in chemotherapy refractory mCRC; subgroup analyses suggested consistent benefit in patients previously treated with anti‐VEGF agents (including bevacizumab). These data provide a clinical rationale for considering fruquintinib as an alternative to bevacizumab in patients with prior anti‐VEGF exposure, offering a novel strategy that may help address resistance to bevacizumab‐based therapy [6]. Additionally, a preclinical study using subcutaneous CRC xenograft models (SW48 and HCT 116) in nude mice demonstrated that the combination of fruquintinib and TAS‐102 outperformed either monotherapy. Specifically, the combination group not only achieved statistically significant reduction in microvessel area but also showed remarkable advantages over single‐agent treatments in suppressing tumor growth in these xenograft models [14]. A multi‐center retrospective study further suggested that TAS 102 combined with fruquintinib has promising clinical efficacy and manageable safety in chemorefractory mCRC, underscoring the need for prospective evaluation of this regimen [15]. Taken together, these studies support the biological and pharmacological rationale for combining fruquintinib and TAS‐102 by integrating complementary anti‐angiogenic and cytotoxic mechanisms and potentially mitigating resistance to prior bevacizumab‐based therapy; however, prospective clinical evidence for this dual regimen in advanced CRC is still lacking.

This prospective, multi‐center, single‐arm, phase II study, as the first prospective trial to evaluate the combination of fruquintinib and TAS‐102, aimed to assess the efficacy and safety of this regimen in patients with refractory mCRC. Our findings are expected to provide preliminary evidence and a foundation for future randomized controlled trials in this setting.

## 2 Results

### 2.1 Patients

Fifty‐three patients from nine clinical research centers in China were screened for eligibility. Finally, 50 patients were enrolled between March 15, 2022, and August 31, 2023. As of the cutoff date, 50 patients were eligible for efficacy and safety assessments. The study flowchart is shown in Figure 1. To characterize the study population, we summarized the patient demographics and baseline clinical characteristics (Table 1). As shown in Table 1, the median age was 60.0 (range: 39–76) years. Eighty‐two percent of patients had left‐sided colon/rectal cancer, and 60% had liver metastasis. Patients with *KRAS* mutations accounted for 50.0% of the participants. For *BRAF* status, 4.0% of patients were *BRAF*‐mutated and 80.0% were *BRAF* wild‐type, with the remaining 16.0% unknown. For MSI status, 86.0% of patients were microsatellite stable/proficient mismatch repair (MSS/pMMR).

**FIGURE 1:**
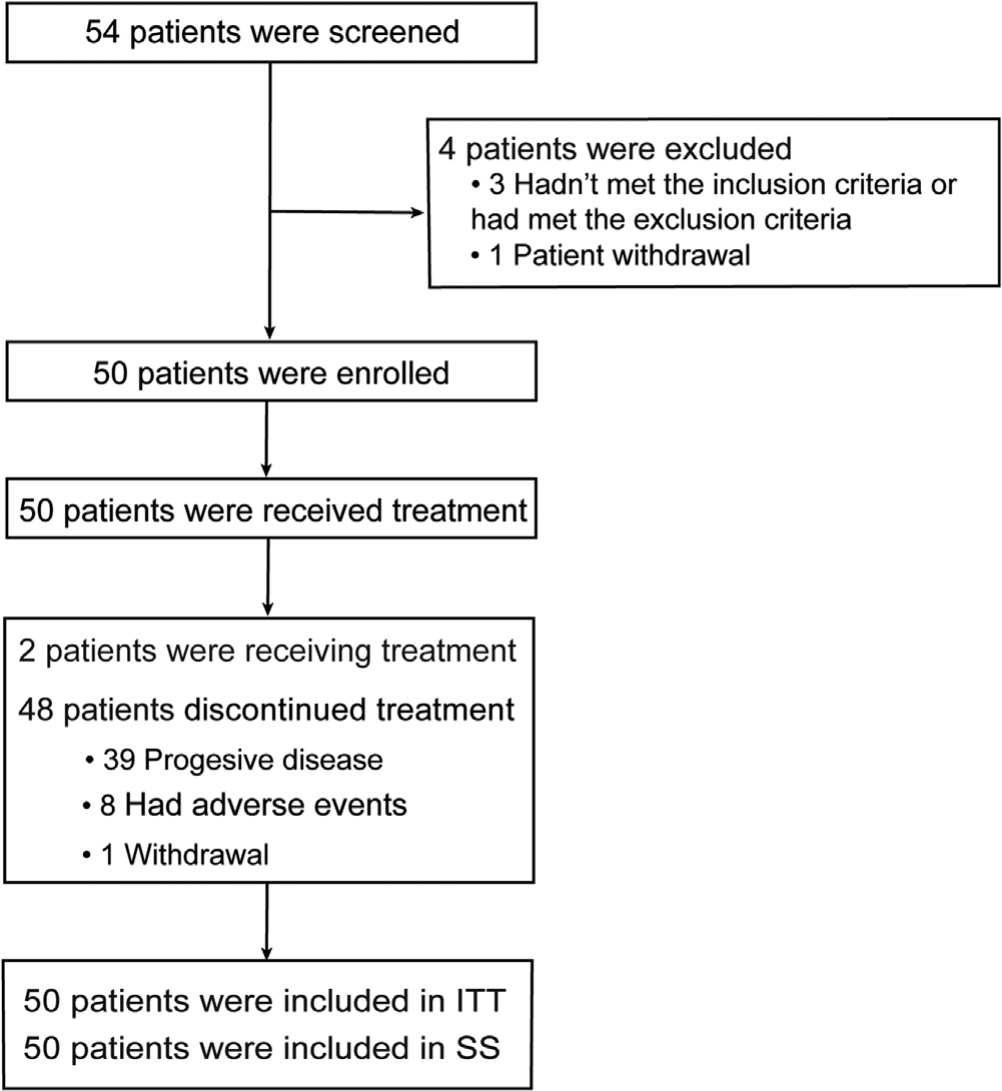
Study flowchart.

**TABLE 1:** Patient demographics and baseline clinical characteristics.

Characteristics	All (*N* = 50)
Age, years, median (range)	60.0 (39, 76)
Sex	
Female	21 (42.0)
Male	29 (58.0)
ECOG performance status	
0	4 (8.0)
1	46 (92.0)
Primary site of disease	
Left‐sided	41 (82.0)
Right‐sided	9 (18.0)
Metastasis	
Liver	30 (60.0)
Lung	30 (60.0)
Peritoneal	9 (18.0)
Multi‐site metastasis	
Yes	34 (68.0)
No	16 (32.0)
*KRAS* mutation	
Wild‐type	18 (36.0)
Mutation	25 (50.0)
Unknown	7 (14.0)
*BRAF* mutation	
Wild‐type	40 (80.0)
Mutation	2 (4.0)
Unknown	8 (16.0)
MSI statue	
MSS/pMMR	43 (86.0)
Unknown	7 (14.0)
Differentiation grade	
Poorly	5 (10.0)
Moderately to poorly	2 (4.0)
Moderately	38 (76.0)
Unknown	5 (10.0)
Number of prior regimens	
2	39 (78.0)
3	9 (18.0)
4	2 (4.0)
Prior anti‐tumor therapy	
Fluoropyrimidine‐based	50 (100.0)
Bevacizumab	44 (88.0)
Cetuximab	13 (26.0)
Histological classification	
Adenocarcinoma	50 (100.0)

*Note*: Data are presented as no. (%) unless otherwise noted. *N*: number of patients.

Abbreviations: ECOG, Eastern Cooperative Oncology Group; MSS, microsatellite stable; pMMR, proficient mismatch repair.

All the patients had previously received at least two antitumor regimens, and 22.0% had received three or more. All patients had undergone fluoropyrimidine‐based therapy, 88.0% had undergone anti‐VEGF therapy (38.0%, 84.0%, or 34.0% received bevacizumab in first‐ or second line therapy or both, respectively), and 26.0% had undergone anti‐EGFR therapy (Table 1). At the time of analysis, only two patients (4.0%) were still receiving treatment, with disease progression being the most common reason for discontinuation.

Collectively, these baseline characteristics reflect a heavily pretreated patient population with poor prognostic features, representative of the refractory mCRC setting in routine clinical practice.

### 2.2 Efficacy

The median follow‐up duration was 25.1 months (IQR: 20.4–29.3). To evaluate the efficacy of the combination regimen, several efficacy‐related indicators were assessed. The median PFS was 6.07 months (95% confidence interval [CI]: 4.07–7.87) (Figure 2A). The 6‐, 9‐, and 12‐month PFS rates were 50.4%, 28.5%, and 18.5%, respectively. The subgroup analysis of PFS showed no significant difference in PFS among all subgroups. The median PFS was comparable between patients with mutated (5.70 months; 95% CI: 2.39–11.25) and wild‐type *KRAS* (5.77 months; 95% CI: 3.70–8.72) (Figure 2B). Patients with peritoneal metastases (median PFS: 6.07 months), lung metastases (median PFS: 6.07 months), or liver metastases (median PFS: 6.07 months), as well as those who received bevacizumab in both first‐line and second‐line therapies (median PFS: 7.48 months), achieved numerically greater PFS benefit compared to the counterpart population.

**FIGURE 2:**
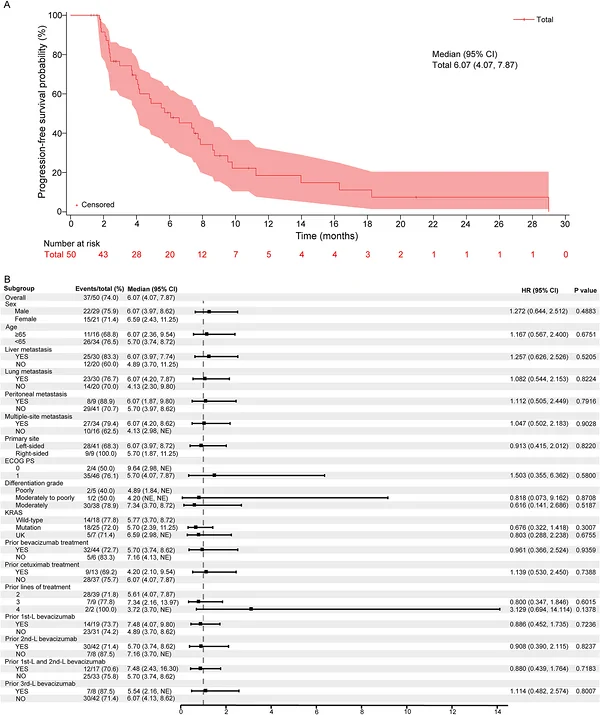
Kaplan–Meier curves of PFS (A) and a forest plot of PFS HRs according to subgroup factors (B). 95% CI, 95% confidence interval; ECOG, Eastern Cooperative Oncology Group; HR, hazard ratio; NE, not estimable; PFS, progression‐free survival.

The median OS was 14.10 months (95% CI: 9.84–23.28) (Figure 3A). The OS rates at 12 and 24 months were 56.9% and 35.9%, respectively. The median OS for patients previously treated with bevacizumab was 14.85 months (95% CI: 8.89–NE), while that for patients without prior bevacizumab treatment was 11.75 months (95% CI: 8.92–NE). The median OS was also comparable between the different subgroups (Figure 3B).

**FIGURE 3:**
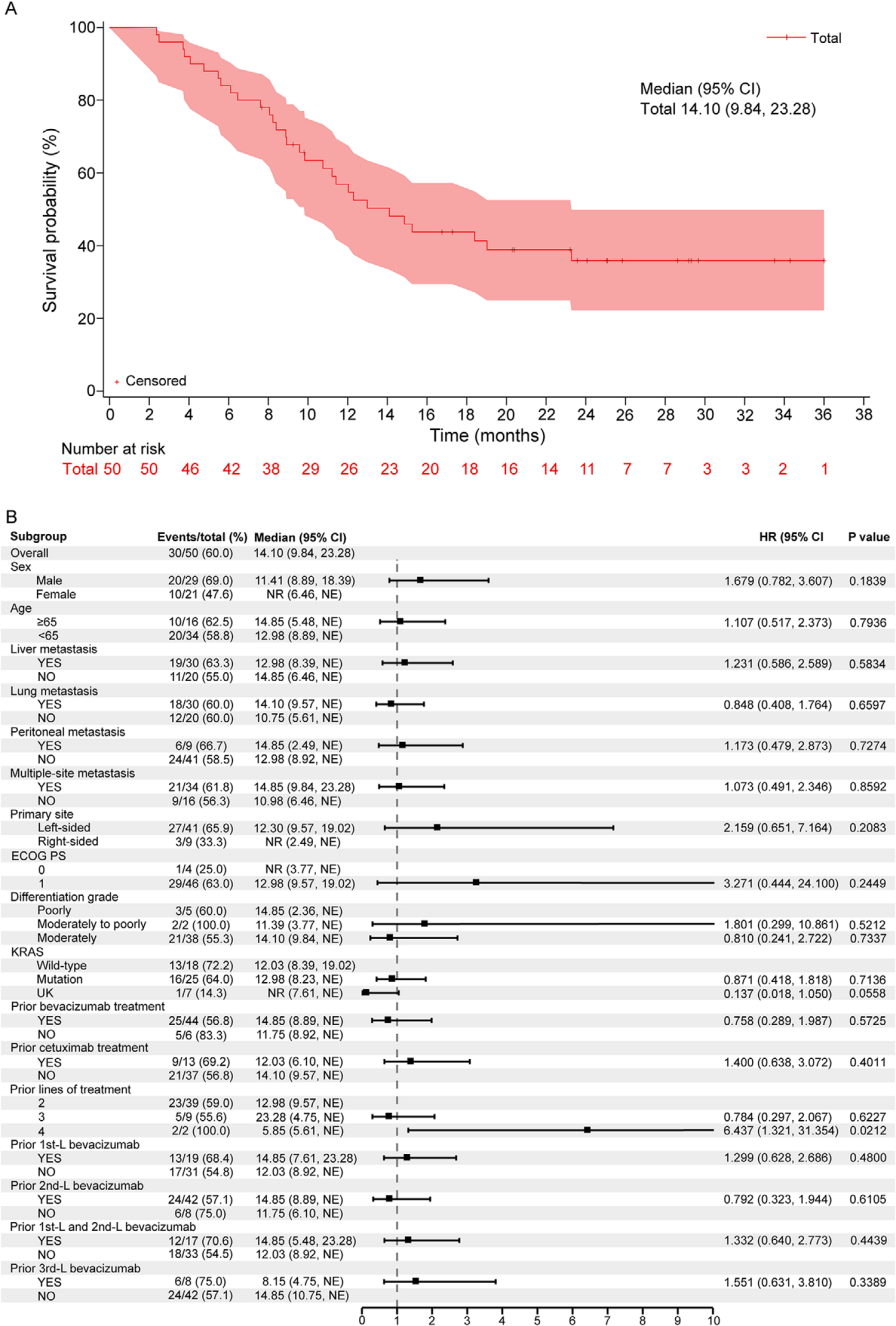
Kaplan–Meier curves of OS (A) and a forest plot of OS HRs according to subgroup factors (B). CI, confidence interval; ECOG, Eastern Cooperative Oncology Group; HR, hazard ratio; NE, not estimable; NR, not reached; OS, overall survival.

No patients achieved complete response (CR), whereas 10.0% (5/50) achieved partial responses (PRs) and 56.0% (28/50) had SDs as the best overall response (BOR). The ORR was 10.0% (95% CI: 3.3–21.8), with a disease control rate (DCR) of 66.0% (95% CI: 51.2–78.8). The detailed efficacy data are presented in Table 2. Comparisons of PFS and OS between the different subgroups are shown in Figures S1–S4.

**TABLE 2:** Summary of responses per investigator assessment.

	All (*N* = 50)
BOR	
PR	5 (10.0)
SD	28 (56.0)
PD	13 (26.0)
NE	4 (8.0)
ORR (95%CI)	10.0 (3.3, 21.8)
DCR (95%CI)	66.0 (51.2, 78.8)
PFS	
Number of events (%)	37 (74.0)
Median (95% CI), months	6.07 (4.07, 7.87)
PFS‐6 (95% CI)	50.4 (34.9, 64.1)
PFS‐9 (95% CI)	28.5 (15.4, 43.1)
PFS‐12 (95% CI)	18.5 (7.7, 32.9)
OS	
Number of events (%)	30 (60.0)
Median (95% CI), months	14.10 (9.84, 23.28)
OS‐12 (95% CI)	56.9 (41.8, 69.4)
OS‐24 (95% CI)	35.9 (22.2, 49.8)

*Note*: Data are presented as no. (%) unless otherwise noted. *N*: number of patients.

Abbreviations: BOR, best overall response; CI, confidence interval; DCR, disease control rate; NE, not evaluable; ORR, overall response rate; OS, overall survival; PD, progressive disease; PFS, progression free survival; PR, partial response; SD, stable disease.

Collectively, these efficacy results indicated that the combination regimen achieved preliminary antitumor activity.

### 2.3 Safety

To evaluate the safety profile of the combination regimen, treatment‑related adverse events (TRAEs) were assessed in all enrolled patients (Table 3). TRAEs were generally manageable. All the patients experienced TRAEs, with 68.0% reporting grade ≥ 3 TRAEs (Table 3). The most common hematological TRAEs (any grade and grades 3–4) included neutrophil count decreased (80.0% and 52.0%, respectively), white blood cell count decreased (68.0% and 24.0%, respectively), anemia (54.0% and 20.0%, respectively), platelet count decreased (46.0% and 8.0%, respectively), and lymphocyte count decreased (38.0% and 8.0%, respectively). The most common nonhematological TRAEs included proteinuria (52.0%), hypoalbuminemia (32.0%), and thyroid stimulating hormone elevated (30%), mainly of grades 1–2. Grade ≥ 3 nonhematological TRAEs that occurred in more than two patients included blood bilirubin elevated (12.0%), proteinuria (4.0%), and hypertriglyceridemia (4.0%). Four patients developed COVID‐19 infection, which was not considered treatment related. Treatment‐related infections included one case of pulmonary fungal infection and one urinary tract infection. No serious AEs (SAEs) were reported, and no treatment‐related deaths occurred. No patients required new or prolonged hospitalization due to TRAEs. A total of seven patients (14.0%) reported TRAEs leading to treatment discontinuation. Seven patients (14.0%) had dose reductions, and 12 patients (24.0%) had dosing delays (Table S1). Granulocyte colony‐stimulating factor (G‐CSF) was administered to 10 patients (20%), mainly in the setting of grade 3–4 neutrophil count decreased. Treatment‐related infections were infrequent (one pulmonary fungal infection and one urinary tract infection), and there were no cases of documented febrile neutropenia. Hand‑foot skin reaction (HFSR) occurred in four patients (8.0%), all of which were grade 1–2 in severity.

**TABLE 3:** Incidence of all grade TRAEs ≥ 10%.

TRAEs, *n* (%)	All (*N* = 50)	
	Any grade	Grade ≥3
Any	50 (100.0)	34 (68.0)
Neutrophil count decreased	40 (80.0)	26 (52.0)
White blood cell count decreased	34 (68.0)	12 (24.0)
Anemia	27 (54.0)	10 (20.0)
Proteinuria	26 (52.0)	2 (4.0)
Platelet count decreased	23 (46.0)	4 (8.0)
Lymphocyte count decreased	19 (38.0)	4 (8.0)
Hypoalbuminemia	16 (32.0)	1 (2.0)
Thyroid stimulating hormone elevated	15 (30.0)	0
Blood bilirubin elevated	13 (26.0)	6 (12.0)
Loss of appetite	11 (22.0)	0
Hypertriglyceridemia	10 (20.0)	2 (4.0)
Diarrhea	9 (18.0)	0
Hypercholesterolemia	9 (18.0)	0
Malaise	8 (16.0)	0
Abdominal pain	8 (16.0)	0
Nausea	6 (12.0)	0
Hypertension	5 (10.0)	1 (2.0)
AST elevated	5 (10.0)	0
Vomiting	5 (10.0)	0
Insomnia	5 (10.0)	0

*Note*: Data are presented as no. (%). *N*: number of patients.

Abbreviations: AST, aspartate aminotransferase; TRAEs, treatment‐related adverse events.

Collectively, these safety data indicate that the combination of fruquintinib and TAS‑102 exhibited a generally manageable and predictable safety profile in this patient population, with no unexpected toxicities or treatment‑related deaths observed.

## 3 Discussion

To the best of our knowledge, this is the first prospective trial assessing the efficacy and safety of fruquintinib combined with TAS‐102 in the later‐line treatment of patients with mCRC. This trial met its primary endpoint, with a median PFS was 6.07 months (95% CI: 4.07–7.87), and a median OS of 14.10 months (95% CI: 9.84–23.28), suggesting that this combination has preliminary antitumor activity with a manageable safety profile.

The 4 mg dose of fruquintinib was selected based on multiple considerations. Although the standard monotherapy dose is 5 mg, dose reduction is a common strategy in combination therapy to ensure safety. A retrospective study of the fruquintinib–TAS‑102 combination showed that the 4 mg dose offered superior efficacy with acceptable safety, supporting its use in this trial [15].

The observed PFS and OS in our study were numerically longer than historical data for fruquintinib or TAS‑102 monotherapy, as well as the TAS‑102–bevacizumab combination (FRESCO: PFS 3.71 months, OS 9.30 months; RECOURSE: PFS 2.0 months, OS 7.1 months; SUNLIGHT: PFS 5.6 months, OS 10.8 months) [6, 8, 9, 12, 13]. However, given the single‑arm design and differences in patient populations, prior treatments, and study methodologies, these cross‑trial comparisons should be interpreted with caution and are provided for contextual reference only.

The potential synergistic interaction between fruquintinib and TAS‑102 is biologically plausible. Fruquintinib suppresses angiogenesis and may improve tumor perfusion and drug delivery, while TAS‑102 exerts cytotoxic effects by inhibiting thymidylate synthase [16, 17, 18, 19, 20]. Preclinical xenograft models have shown that the combination significantly inhibited tumor growth, with activity superior to either agent alone [14]. These complementary mechanisms support further clinical evaluation.

In exploratory subgroup analyses, PFS was comparable between patients with *RAS* mutations and those with wild‑type *RAS*. Notably, patients with prior bevacizumab exposure (88.0%) appeared to derive numerical OS benefit, contrasting with a post hoc analysis of the SUNLIGHT study in which continuous bevacizumab therapy was associated with less benefit from TAS‑102 plus bevacizumab [21]. Fruquintinib's broader VEGFR‑1/2/3 inhibition may overcome resistance pathways triggered by bevacizumab [22, 23, 24, 25]. However, these findings are descriptive and hypothesis‑generating, requiring validation in larger trials.

Compared with combinations involving bevacizumab or other intravenous drugs, another advantage of the fruquintinib and TAS‐102 combination is that both drugs are oral formulations, which allows treatment plans to be executed in an outpatient setting, eliminates the need for hospitalization and reduces both the hospital inpatient burden and patients' healthcare costs.

All patients experienced TRAEs, and 68% developed grade ≥ 3 events, underscoring the need for close monitoring and proactive supportive care. The most prominent toxicity was neutropenia, with grade ≥ 3 neutrophil count decreased occurring in 52.0% of patients—consistent with the 52% incidence reported in the SUNLIGHT trial (TAS‑102 plus bevacizumab) and 46.9% in a retrospective fruquintinib–TAS‑102 study [12, 21, 26, 27, 28, 29, 30]. The clinical impact of neutropenia was limited: G‑CSF was administered to 10 patients (20.0%), and dose reductions or suspensions due to neutropenia allowed continuation of treatment without febrile neutropenia, infection‑related SAEs, or hospitalization. Except for neutropenia, the incidences of other grade ≥ 3 AEs were comparable to those reported with either agent alone, and no treatment‑related deaths or new safety signals were observed. Regarding the clinical management of myelosuppression, alternative dosing strategies have been explored to mitigate this toxicity. Notably, the phase III ROBITS study evaluated a biweekly TAS‑102 schedule combined with bevacizumab but failed to demonstrate OS benefit, highlighting the challenge of balancing toxicity reduction with preserved efficacy [13]. Given this, future investigations should explore optimized schedules—including biweekly fruquintinib combined with TAS‑102—as a potential strategy to improve the therapeutic index of this VEGF inhibitor–TAS‑102 combination. Overall, no new safety signals emerged, and the safety profile of this fruquintinib–TAS‑102 regimen is considered acceptable and clinically manageable with appropriate supportive care.

This study has several limitations that warrant cautious interpretation of the findings. First and foremost, this was an open‐label, non‐randomized, single‐arm phase II clinical study. The absence of a concurrent control arm precludes definitive conclusions regarding the true incremental benefit of the combination or direct comparisons to standard care. Consequently, the observed efficacy, even if numerically favorable relative to historical data, may be subject to unmeasured confounding and selection bias. Second, although the planned sample size was achieved (*N* = 50), the final analysis was based on fewer PFS events than anticipated in the original statistical design (37 vs. 43). Together with the modest sample size and missing biomarker information in a proportion of patients, this may have reduced the statistical information available and limited the interpretability and generalizability of the exploratory subgroup analyses. Subgroup findings, such as the potential benefit in bevacizumab‐pretreated patients, comparable efficacy between patients with *RAS* mutations and those with wild‐type *RAS*, must be interpreted as hypothesis‐generating and require validation in larger trials. Third, the heterogeneity of the refractory patient population is a factor. While most patients received two prior regimens, the inclusion of some patients who were on the fourth or later lines of treatment, and variability in prior exposure to agents like cetuximab, could have introduced further heterogeneity. Furthermore, interpretation of OS in this study is limited by the absence of systematic documentation of post‐progression therapies. As such, the observed OS outcomes may have been influenced by these uncollected post‐progression treatments and should therefore be considered descriptive and hypothesis‐generating. Fourth, the 4 mg starting dose of fruquintinib was selected based on empirical clinical experience and retrospective data, rather than on formal phase I dose‑finding studies specifically for this combination. Although the observed safety profile and tolerability suggest that this dose is feasible in the current patient population, the lack of systematic dose‑escalation data for the fruquintinib–TAS‑102 combination may limit the ability to determine the optimal biological dose. Therefore, while this study successfully established the clinical activity and acceptable safety profile of the fruquintinib‐TAS‐102 combination, further confirmation of the efficacy and any potential synergistic effect is essential in future large‐scale, randomized, controlled Phase III clinical studies.

In conclusion, fruquintinib in combination with TAS‐102 demonstrated preliminary anti‐tumor activity in patients with mCRC who had previously experienced treatment failure with at least two standard therapies. The fruquintinib–TAS‐102 combination regimen demonstrated a manageable safety profile, but requires close monitoring for hematologic toxicities. Future large‐sample phase III clinical studies should further validate the efficacy of fruquintinib combined with TAS‐102 in this setting.

## 4 Materials and Methods

### 4.1 Study Design and Patients

This phase II, single‐arm, multi‐center trial prospectively recruited adults (aged 18–75) with unresectable, histologically confirmed colon or rectal adenocarcinoma. Eligible candidates had an Eastern Cooperative Oncology Group (ECOG) score of 0–1 and were refractory to at least two standard regimens, such as those containing irinotecan, oxaliplatin, and fluoropyrimidine. Treatment failure was defined as progressive disease (PD) or unacceptable toxicity during treatment or within 3 months after the last treatment. Each line of therapy must have included at least one cycle of one or more chemotherapeutic agents. Patients with recurrence or metastasis during or within 6 months of completion of adjuvant/neoadjuvant therapy were considered to have experienced treatment failure to the respective first‐line treatment. Prior treatment with chemotherapy combined with cetuximab or bevacizumab was permitted, but patients who had previously received fruquintinib or other oral VEGF receptor tyrosine kinase inhibitors (such as apatinib, regorafenib, and anlotinib) or TAS‐102 were excluded. Enrolled patients had a life expectancy of ≥ 3 months with adequate organ function.

Prior to the initiation of the trial, the study protocol had been reviewed and approved by the Ethics Committee of each site, and written informed consent was obtained from each patient prior to their enrollment. The trial was performed in strict compliance with the ethical principles outlined in the Declaration of Helsinki as well as the Good Clinical Practice guidelines issued by the International Council for Harmonization. The trial was registered at clinicaltrials.org (NCT05004831).

### 4.2 Treatments

Eligible patients were treated with a combination of fruquintinib and TAS‐102. Fruquintinib was administered orally once daily for 3 weeks, followed by a 1‐week break, at a dosage of 4 mg per day (4‐week cycles). TAS‐102 was taken orally twice a day on Days 1 to 5 and Days 8 to 12 of each cycle at a dose of 35 mg/m^2^ each time. Treatment continued until PD, unacceptable toxicity, or withdrawal of consent. The above dosing regimen was adjusted as needed on the basis of the patient's tolerance. The principle for dose adjustment should follow the sequence of treatment interruption prior to dose reduction. Following treatment suspension, resumption at the original dose was permitted if adverse reactions resolved to ≤ Grade 1 within 1 week; if resolution was achieved within 2 weeks, dose adjustment was advised under physician supervision. In the case of fruquintinib, adjustment to 3 mg once daily was recommended, and permanent discontinuation was necessary if intolerance continued at this dose level. With respect to TAS‐102, up to 3 dose reductions were allowed, and the minimum dose was 20 mg/m^2^ twice daily. Detailed dose adjustments are referred to in the Supporting Information Methods.

### 4.3 Endpoints and Assessments

The primary endpoint of this trial was PFS, which was defined as the time interval from enrollment to PD or death from any cause, whichever came first. The secondary endpoints included OS, ORR, and DCR, which were assessed according to the RECIST 1.1 criteria [31] and safety. OS was defined as the time interval from enrollment to death from any cause. The ORR was defined as the proportion of patients whose BOR was CR or PR. The DCR was defined as the percentage of patients with a BOR of CR, PR, or stable disease (SD).

Clinical evaluations and CT scans were carried out at baseline and every two treatment cycles until confirmed PD, death, or withdrawal of consent (whichever occurred first). Response was assessed by the local investigator in accordance with the RECIST version 1.1. Patients were contacted every 8 weeks to assess their vital status until death or trial completion. Safety assessments were regularly carried out throughout treatment and 30 days post treatment. Incidence and severity of AEs were graded using the National Cancer Institute Common Terminology Criteria for Adverse Events (CTCAE) version 5.0.

### 4.4 Statistical Analysis

Sample size calculations were based on a one‐sample log‐rank test, with the assumptions of a one‐sided significance level of 0.05, a power of 80%, and a historical median PFS of 3.7 months for fruquintinib monotherapy and an anticipated median PFS of 5.5 months for combination therapy. This required 48 patients (43 events) to achieve a power of 80% to demonstrate that the combination therapy was significantly superior to the historical control (fruquintinib monotherapy). Accounting for a 10% dropout rate, the calculated sample size required for enrollment was 54 patients.

Efficacy outcomes were assessed on the basis of the intention‐to‐treat (ITT) population (primary analysis and subgroup analyses), which included all patients who were successfully enrolled. The safety set comprised all enrolled patients who had been treated with at least one dose of study medication. Safety analysis was performed for the safety set. Categorical variables are presented as case counts and corresponding percentages. For continuous variables, descriptive statistics are reported, encompassing the number of observations, as well as the median, lower quartile, upper quartile, minimum, and maximum values. The median follow‐up time was estimated via the reverse Kaplan–Meier method. Categorical outcomes (e.g., ORR and DCR) are presented as percentages and two‐sided 95% CIs, which were computed using the Clopper‒Pearson method. Time‐to‐event outcomes (e.g., PFS and OS) were estimated using the Kaplan‒Meier method, and the corresponding 95% CIs were calculated using the log‐log transformation approach. Survival curves were compared using the nonparametric log‐rank test. Univariate Cox regression analyses were performed to identify clinical factors associated with PFS and OS. Statistical significance was defined as a two‐sided *p* value < 0.05 or 95% CI for hazard ratio (HR) that does not include 1. Statistical analyses were conducted with SAS software (Version 9.4).

## Author Contributions

Jianjun Peng, Jianting Long, Haibo Zhang, Chuangqi Chen, and Shirong Cai contributed to the conception and design of the study. Jianjun Peng, Yong Li, Ling Huang, Jianhua Liu, Jianjun Xiao, Derong Xie, Wei Wang, Zongcheng Zhang, Chengyin Weng, Xiaoshu Chai, Huifen Li, Xiaomei Jiang, Yan Qian, Risheng Zhao, Sile Chen, Zhimin Jiang, Lingdan Le, Xueqin Li, Jianting Long, Haibo Zhang, Chuangqi Chen, and Shirong Cai were involved in the acquisition, analysis, and interpretation of the data. Jianjun Peng supervised the study. Jianjun Peng, Yong Li, Ling Huang, Jianhua Liu, and Jianjun Xiao wrote and revised the manuscript. All authors have read and approved the final manuscript.

## Funding

The trial was funded by HUTCHMED (China) Limited and Qilu Pharmaceutical Co., Ltd., which provided investigational products and related study materials but were not involved in study design, data collection, and data analysis. Medical writing and editorial assistance were provided by a professional medical communications agency, as non‐author contributors, and were funded by HUTCHMED (China) Limited. The authors retained full control over the manuscript content and the decision to submit for publication. HUTCHMED was given the opportunity to review the manuscript prior to submission but had no role in the writing of the manuscript or in the decision to submit.

## Ethics Statement

Prior to the initiation of the trial, the study protocol had been reviewed and approved by the Ethics Committee of the First Affiliated Hospital of Sun Yat‐sen University (approval No. 2021‐573), and written informed consent was obtained from each patient prior to their enrollment. The trial was performed in strict compliance with the ethical principles outlined in the Declaration of Helsinki as well as the Good Clinical Practice guidelines issued by the International Council for Harmonization.

## Conflicts of Interest

The authors declare no conflicts of interest.

## Supporting information




**Supporting File**: mco271070‐sup‐0001‐SuppMat.docx

## Data Availability

All data generated in this study are available within the article and its Supporting Information. The corresponding author will provide the datasets used in this study upon receipt of a reasonable request. The corresponding authors had full access to all study data and take full responsibility for the integrity and accuracy of the data and all analyses.
