# Supplementary material for: Efficacy and Safety of Fruquintinib Plus TAS‐102 in Patients With Previously Treated Metastatic Colorectal Cancer: Results From a Single‐Arm, Multi‐Center, Phase II Study
Source: MedComm (2020). 2026 Oct 13;7(10):e71070. doi: 10.1002/mco2.71070 (PMC13659284; doi:10.1002/mco2.71070)
Supplement: Supplementary file 1 [file MCO2-7-e71070-s001.docx]

**Efficacy and Safety of Fruquintinib plus TAS-102 as a Third-Line Treatment in Patients with Metastatic Colorectal Cancer: Results from a Single-Arm, Multicenter, Phase Ⅱ Study**

Jianjun Peng^1#^*, Yong Li^2#^, Ling Huang^3#^, Jianhua Liu^3#^, Jianjun Xiao^4#^, Derong Xie^5^, Wei Wang^6^, Zongcheng Zhang^7^, Chengyin Weng^8^, Xiaoshu Chai^9^, Huifen Li^4^, Xiaomei Jiang^4^, Yan Qian^1^, Risheng Zhao^1^, Sile Chen^1^, Zhimin Jiang^5^, Lingdan Le^5^, Xueqin Li^10^, Jianting Long^1^*, Haibo Zhang^9^*, Chuangqi Chen^1^*, Shirong Cai^1^*

^1^The First Affiliated Hospital of Sun Yat-sen University, Guangzhou, P. R. China;

^2^Guangdong Provincial Hospital of Chinese Medicine, Zhuhai, P. R. China;

^3^Guangdong Provincial People’s Hospital, Guangdong Academy of Medical Sciences, Southern Medical University, Guangzhou, P. R. China;

^4^Zhongshan City People's Hospital, Zhongshan, P. R. China;

^5^Sun Yat-sen Memorial Hospital of Sun Yat-sen University, Guangzhou, P. R. China;

^6^The First People's Hospital of Foshan, Foshan, P. R. China;

^7^Liwan Central Hospital of Guangzhou, Guangzhou, P. R. China;

^8^Guangzhou First People's Hospital, Guangzhou, P. R. China;

^9^Guangdong Provincial Hospital of Chinese Medicine, Guangzhou, P. R. China;

^10^Shenshan Medical Center, Memorial Hospital of Sun Yat-sen University, Shanwei, P. R. China.

^#^**Joint first authors.**

***These authors jointly supervised this work.**

**Corresponding authors:**

**Jianjun Peng**, Department of Gastrointestinal Surgery, The First Affiliated Hospital of Sun Yat-sen University, No. 58 Zhongshan Er Road, Guangzhou 510080, Guangdong, P. R. China. E-mail: pjianj@mail.sysu.edu.cn.

**Jianting Long**, Department of Oncology, The First Affiliated Hospital of Sun Yat-sen University, No. 58 Zhongshan Er Road, Guangzhou 510080, Guangdong, P. R. China. E-mail: longjt2@mail.sysu.edu.cn.

**Haibo Zhang**, Department of Oncology, Guangdong Provincial Hospital of Chinese Medicine, No. 111 Dade Road, Guangzhou 510120, Guangdong, P. R. China. E-mail: haibozh@gzucm.edu.cn.

**Chuangqi Chen**, Department of Gastrointestinal Surgery, The First Affiliated Hospital of Sun Yat-sen University, No. 58 Zhongshan Er Road, Guangzhou 510080, Guangdong, P. R. China. E-mail: chenchqi@mail.sysu.edu.cn.

**Shirong Cai**, Department of Gastrointestinal Surgery, The First Affiliated Hospital of Sun Yat-sen University, No. 58 Zhongshan Er Road, Guangzhou 510080, Guangdong, P. R. China. E-mail: caishr@mail.sysu.edu.cn.

Supplementary Figures

**Figure S1.** Kaplan-Meier curves of PFS stratified by (A) age; (B) sex; (C) ECOG; (D) primary site; (E) liver metastasis; (F) lung metastasis; (G) peritoneal metastasis and (H) multiple-site metastasis. PFS, progression-free survival.

**Figure S2.** Kaplan-Meier curves of PFS stratified by (A) differentiation grade; (B) KRAS; (C) prior bevacizumab treatment; (D) prior cetuximab treatment; (E) prior lines of treatment; (F) prior first-line bevacizumab treatment; (G) prior second-line bevacizumab treatment; (H) prior first-line and second-line bevacizumab treatment and (I) prior third-line bevacizumab treatment. PFS, progression-free survival.

**Figure S3.** Kaplan-Meier curves of OS stratified by (A) age; (B) sex; (C) ECOG; (D) primary site; (E) liver metastasis; (F) lung metastasis; (G) peritoneal metastasis; and (H) multiple-site metastasis. OS, overall survival.

**Figure S4.** Kaplan-Meier curves of OS stratified by (A) differentiation grade; (B) KRAS; (C) prior bevacizumab treatment; (D) prior cetuximab treatment; (E) prior lines of treatment; (F) prior first-line bevacizumab treatment; (G) prior second-line bevacizumab treatment; (H) prior first-line and second-line bevacizumab treatment and (I) prior third-line bevacizumab treatment. OS, overall survival.

Supplementary Table

**Table S1.** TRAEs leading to dose modification and treatment discontinuation.

Supplementary Methods

Dose adjustment

**Figure S1.**

**Figure S2.**

**Figure S3.**

**Figure S4.**

**Table S1.** TRAEs leading to dose modification and treatment discontinuation.

| **TRAEs** | **All (N = 50)** |
| --- | --- |
| TRAEs leading to dose delay | |
| All | 12 (24.0) |
| Neutrophil count decreased | 7 (14.0) |
| Hand-foot skin reaction | 1 (2.0) |
| Anemia | 1 (2.0) |
| White blood cell count decreased | 1 (2.0) |
| Proteinuria | 1 (2.0) |
| Hypoalbuminemia | 1 (2.0) |
| Abdominal pain | 1 (2.0) |
| Oral ulceration | 1 (2.0) |
| Hypertension | 1 (2.0) |
| Fever | 1 (2.0) |
| Urinary tract infection | 1 (2.0) |
| Serum creatinine increased | 1 (2.0) |
| TRAEs leading to dose reduction | |
| All | 7 (14.0) |
| Neutrophil count decreased | 4 (8.0) |
| White blood cell count decreased | 2 (4.0) |
| Platelet count decreased | 1 (2.0) |
| Proteinuria | 1 (2.0) |
| Oral ulceration | 1 (2.0) |
| Stoma site hemorrhage | 1 (2.0) |
| TRAEs leading to treatment discontinuation | |
| All | 7 (14.0) |
| Proteinuria | 4 (8.0) |
| Stoma site hemorrhage | 2 (4.0) |
| Hypoalbuminemia | 1 (2.0) |
| Platelet count decreased | 1 (2.0) |
| Abdominal pain | 1 (2.0) |

*Note*: Data are presented as no. (%). N: number of patients.

Abbreviations: TRAEs, treatment-related adverse events.

Supplementary Methods

**Dose Adjustment**

1. Principles of Fruquintinib Dose Adjustment

Drug-related toxic and side effects occurring in patients during the trial will be graded in accordance with the NCI CTCAE Version 5.0. Physicians will closely monitor patients during medication administration and adjust the dosage of fruquintinib based on the individual safety and tolerability of patients, including temporary suspension of medication, dose reduction, or permanent discontinuation. Dose adjustment will follow the principle of suspending medication first and then reducing the dose.

- After temporary suspension of medication, if the adverse reaction resolves to ≤ Grade 1 within 1 week, continue administration at the original dose;
- If the adverse reaction resolves to ≤ Grade 1 within 2 weeks, it is recommended to adjust the dose to 3 mg per day (3 capsules, each containing 1 mg of fruquintinib) under the guidance of a physician; if 3 mg per day is still not tolerable, permanently discontinue the medication.

2. Detailed Operational Rules for Fruquintinib Dose Adjustment

The severity of adverse reactions is graded using the NCI CTCAE Version 5.0. The general principles for fruquintinib dose adjustment of are shown in Table 1.

**Table 1.** Principles for Fruquintinib Dose Adjustment in the Treatment of mCRC.

| **Dose Adjustment Regimen** | **Applicable Adverse Reaction Occurrences** |
| --- | --- |
| Suspension | - Grade 2 bleeding at any site - Grade 2 hand-foot skin reaction and recurrent oral mucositis - Grade 2 thrombocytopenia (50~75×10^9^/L) - 24-hour urinary protein quantification ≥ 2.0 g - All Grade 3 or 4 adverse reactions (excluding adverse reactions requiring permanent discontinuation) |
| Reduction | - Adverse reaction resolves to ≤ Grade 1 within 2 weeks after temporary suspension of medication |
| Permanent discontinuation | - Grade 3 or above bleeding - Gastrointestinal perforation, clinically significant wound dehiscence requiring treatment, fistula, nephrotic syndrome, or hypertensive crisis - Grade 4 liver dysfunction or injury (transaminase > 20 times the upper limit of normal value) - 3 mg per day is still not tolerable - Adverse reaction does not resolve to ≤Grade 1 within more than 2 weeks after temporary suspension of medication |

3. Detailed Operational Rules for TAS-102Dose Adjustment

The dose of TAS-102 is adjusted based on the safety and tolerability. A maximum of 3 dose reductions is allowed, with the minimum dose of 20 mg/m² per dose, twice daily. Once the dose is reduced, it won’t be increased.

If hematological and/or non-hematological toxicity occurs, corresponding measures will be taken in accordance with the suspension, resumption, and dose reduction criteria in Tables 2 and 3. The suspension, resumption, and dose reduction criteria based on body surface area (BSA) are referred to the package insert.

**Table 2.** Dose Adjustment for Myelosuppression-Related Hematological Toxicity.

| **Parameter** | **Suspension Criteria** | **Resumption Criteria** |
| --- | --- | --- |
| Neutrophils | ＜0.5×10^9^/L | ≥1.5×10^9^/L |
| Platelet count | ＜50×10^9^/L | ≥75×10^9^/L |

Note: Regardless of whether the suspension criteria are met, the resumption criteria apply to the start of the next course of treatment for all patients.

**Table 3.** Dose Adjustment for Hematological and Non-hematological Adverse Reactions.

| **Adverse Reactions** | **Recommended Dose Adjustment** |
| --- | --- |
| - Febrile neutropenia - Grade 4 neutropenia (＜0.5×10⁹/L) or thrombocytopenia (＜25×10⁹/L) that delays the next course of treatment by more than one week. - Grade 3 or 4 non-hematological adverse reactions; excluding Grade 3 nausea and/or vomiting controllable with antiemetic therapy, or Grade 3 diarrhea responsive to antidiarrheal drugs. | - Suspend until toxicity resolves to Grade 1 or baseline level. When restarting treatment, reduce the dose by 5 mg/m² per dose from the previous dose level. - A maximum of dose reduction to the minimum dose of 20 mg/m² per dose, twice daily, is allowed. - No dose increase is permitted after dose reduction. |
